# Revision of the genus *Buchneria* (Bryozoa, Cheilostomata) from Japan

**DOI:** 10.3897/zookeys.241.3175

**Published:** 2012-11-12

**Authors:** Masato Hirose

**Affiliations:** 1 National Museum of Nature and Science Tokyo, 4-1-1, Amakubo, Tsukuba, Ibaraki 305-0005, Japan

**Keywords:** *Buchneria dofleini*, *Buchneria teres*, *Buchneria rhomboidalis*, *Buchneria variabilis*, new combination, syno-nymy, distribution, Sagami Bay, Akkeshi

## Abstract

*Buchneria dofleini* (Buchner, 1924), type species of *Buchneria* Harmer, 1957,was first described from material collected in 1904–1905 from Sagami Bay, Japan, but the type specimens had not been reexamined since the original description. In this study, I examined specimens of *Buchneria* from historical collections and material recently collected near Akkeshi, Hokkaido, Japan. Three *Buchneria* species were detected, two from Sagami Bay that Ortmann (1890) had placed in *Escharoides*, and one from Akkeshi that Androsova (1958) had described as *Porella variabilis*. I concluded that *Buchneria dofleini* is a junior synonym of *Escharoides teres* Ortmann, 1890; selected a lectotype for *Escharoides teres* among Ortmann’s syntypes; and established the new combination *Buchneria teres* (Ortmann, 1890), which becomes the type species of *Buchneria*. I also established the new combination *Buchneria rhomboidalis* (Ortmann, 1890) and selected a lectotype among Ortmann’s syntypes. *Porella variabilis* is transferred to *Buchneria* establishing the new combination *Buchneria variabilis* (Androsova, 1958). Here the three new combinations are redescribed and a key to the Japanese *Buchneria* species is provided. Finally, I transferred *Buchneria* to Bryocryptellidae on the basis of ovicell and orifice morphology. Therefore, *Buchneria* now includes a total of three species; *Buchneria sinuata* Harmer, 1957, a species from Indonesia that has hitherto been placed in this genus, is almost certainly not congeneric with other *Buchneria*. As far as is now known, *Buchneria* is endemic to northern Japan and the northern Sea of Japan.

## Introduction

Harmer (1957) introduced the cheilostome bryozoan genus *Buchneria* for species with erect colonies, and chose the deep sea species *Palmicellaria dofleini* as type, which was described by Buchner (1924) from Sagami Bay, Japan. According to Harmer’s description, zooids in species of *Buchneria* have the proximal margin of the orifice with a broad sinus or nearly straight, with a small suboral avicularium at the edge of the peristome, and have only a few pores in the frontal shield. These characters, however, also match *Escharoides teres* and *Escharoides rhomboidalis*, both described by Ortmann (1890) from Sagami Bay, although Ortmann’s (1890) limited descriptions and simplified illustrations are inadequate for taxonomic assessment.

The status of *Buchneria* has not been evaluated subsequent to Harmer (1957). In this study, I reexamined type specimens established by Buchner (1924) and Ortmann (1890) for species of *Buchneria* and morphologically similar genera. I examined relevant material collected in Japan over the past approximately 130 years, as well as material obtained through my own collecting efforts. I review, describe, and illustrate the known species of *Buchneria*.

### Materials and methods. Material examined

I examined specimens from Sagami Bay and surrounding areas collected by Ludwig Döderlein (1880–1881), Franz Doflein and Karl Haberer (1904–1905), Emperor Showa (1918–1971), and most recently by the National Museum of Nature and Science Tokyo (2001–2005); see National Museum of Nature and Science (2007), Hirose (2010), and Spencer Jones et al. (2011) for historical overviews. This material is housed in Musée Zoologique Strasbourg (MZS), Zoologische Staatssammlung München (ZSM), Senckenberg Forschungsinstitut und Naturmuseum in Frankfurt, Germany (SMF; material on loan there from ZSM), and the National Museum of Nature and Science Tokyo (NMST), now located in Tsukuba (see Supplementary Table 1).

**Figure 1.**
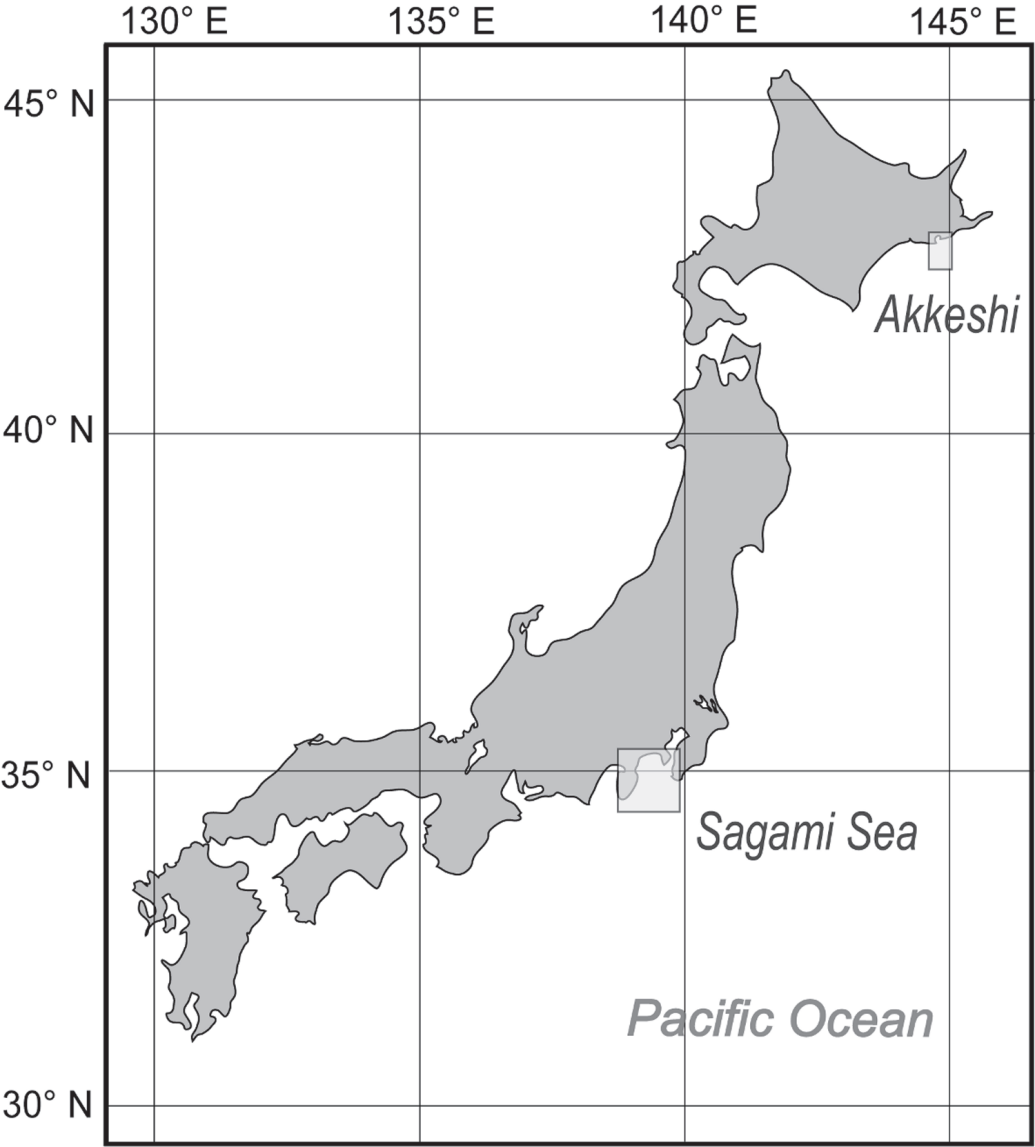
Map showing the areas in Japan where species of *Buchneria* were collected.

The author collected additional specimens from Sagami Bay by dredge from RV *Tansei-maru* (Japan Agency for Marine-Earth Science and Technology, JAMSTEC) and research boat *Rinkai-maru* (Misaki Marine Biological Station, The University of Tokyo) in November 2007 and February 2012, and outside Akkeshi Bay, Hokkaido, in July 2010 and 2011, by dredge from research boat *Misago-maru* (Akkeshi Marine Station, Hokkaido University) (Fig. 1).

### Preparation and observation of specimens

Specimens were observed by light microscope and scanning electron microscope (SEM). For SEM observation, part of each specimen was removed, soaked in a sodium hypochlorite solution to remove the soft tissue, rinsed in water, air dried, and mounted with double-sided adhesive tape or silver paste on an aluminum SEM stub. At Hokkaido University, mounted specimens were coated with Au in a Hitachi E-1030 sputter-coater and observed with a Hitachi S-3000N SEM at 15 kV accelerating voltage. At the SMF, specimens were coated with Pt-Pd and observed with a CamScan SEM. At ZSM, specimens were coated with Au in a POLARON SEM Coating System and observed with a LEO 1430VP SEM at 15 kV accelerating voltage. Fragments removed from specimens in the various collections, prepared and examined by SEM, and subsequently deposited in NMST are indicated in the text by the designation ‘NSMT Te’ (see Supplementary Table 1).

Measurements were taken from SEM images with ImageJ 1.37v software (Image Processing and Analysis in Java, Wayne Rasband, National Institutes of Health, USA; http://rsb.info.nih.gov/ij/ ). Measurements in the text are presented in millimeters, as ranges followed in parentheses by the mean and standard deviation. Sample sizes for measurements were n = 4–82, generally from more than one colony. Abbreviations used for measurements are as follows; ZL, zooid length; ZW, zooid width; OrL, orifice length; OrW, orifice width; AvL, suboral avicularium length; AvW, suboral avicularium width; OvL, ovicell length; OvW, ovicell width.

## Taxonomy

### Order Cheilostomata Busk, 1852. Suborder Neocheilostomina d’Hondt, 1985. Infraorder Ascophorina Levinsen, 1909. Superfamily Lepralielloidea Vigneaux, 1949. Family Bryocryptellidae Vigneaux, 1949

#### Genus Buchneria

Harmer, 1957

http://species-id.net/wiki/Buchneria

##### Type species.

*Palmicellaria dofleini* Buchner, 1924 by original designation by Harmer (1957: 876) (= *Escharoides teres* Ortmann, 1890).

##### Diagnosis.

**Figure 2.**
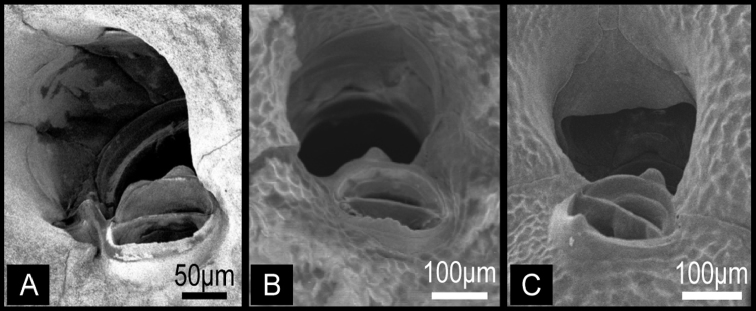
Orifices of three *Buchneria* species showing the small tooth distal to the suboral avicularium. **A** *Buchneria teres* **B** *Buchneria rhomboidalis* **C** *Buchneria variabilis*.

**Figure 3.**
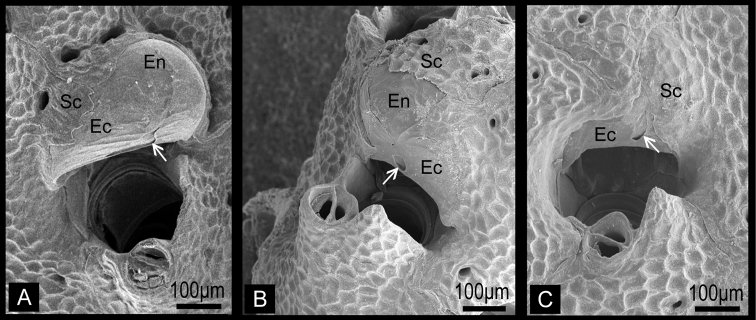
Ovicells of *Buchneria teres* showing various stages of development. **A** Younger stage ooecium showing smooth surface of endooecium and ectooecium with less secondary calcification**B** Ooecium started covered by tessellated secondary calcification from neighboring zooids**C** Ooecium almost covered by the secondary calcification with showing endooecium through the small proximal membranous window at ectooecium. Ec, ectooecium; En, endooecium; Sc, secondary calcification. Arrows indicate the proximal membranous window.

Colony erect, rigid, dichotomously or irregularly branching: branches cylindrical, flattened, or plate-like, fan shaped. Zooidal frontal shield uniformly tessellated, with a few areolar pores near margin or offset centrally in secondarily calcified wall. Orifice deeply immersed, without teeth on distal periphery, without lyrula or condyles, slightly concave or straight proximally; oral spines absent. Secondary orifice at colony surface cormidial, formed by contributions of secondary calcification from distal and lateral zooids. Suboral avicularium lies at proximal margin of secondary orifice, directed proximally or laterally, sometimes enlarged and occupying about half of frontal shield; small, conical tooth associated with avicularium projecting into secondary orifice (Fig. 2). Mandible of the suboral avicularium semicircular or spatulate, but never acute. Vicarious and other frontal avicularia absent. Ovicell globose, acleithral, and is produced by the distal zooid (Fig. 3). Both the endooecium and ectooecium are calcified. Endooecium is completely calcified, whereas ectooecium is not completely covering the endooecium (Fig. 3A, B). Immediately after formation, the ectooecium is then partially covered by the secondary calcification that is coming from the distal and neighbour zooids (Fig. 3B). Finally, the secondary calcification covers most of the ectooecium in the old parts of the colony, but a small area of proximal margin remains uncovered (Fig. 3C). Small basal pore chambers present.

##### Remarks.

Harmer (1957) defined *Buchneria* as follows: colony erect, not jointed; large spatulate avicularia present; zooids with a “sinuate” or nearly straight proximal margin of the orifice (the term ‘sinuate’ appears to be misapplied to the evenly concave proximal margin in *Buchneria sinuata*; perhaps Harmer intended the meaning as ‘having a sinus’); small, acute suboral or lateral avicularia on the edge of the secondary orifice; few frontal pores; and a hyperstomial ovicell with an imperforate central tabula. His generic diagnosis, however, largely derives from *Buchneria sinuata* Harmer, 1957 from Indonesia. This species is similar to the three *Buchneria* species treated herein in having erect colony form, few frontal or marginal pores, and a deeply immersed primary orifice. However, it differs substantially from them in having large, spatulate frontal avicularia; hyperstomial ovicells with an imperforate central tabula lacking secondary calcification; and a laterally placed, acute oral avicularium (Harmer, 1957: plate LIV, fig. 19). *Buchneria sinuata*, therefore, has currently been thougth to belong in another genus, perhaps *Osthimosia* Jullien, 1888 (Gordon, 1984). Gordon (1984) noted several similarities between *Osthimosia virgula* and nominal *Buchneria sinuata* (e.g., broad orificial sinus, a lateral-oral avicularium on peristome, and spatulate frontal avicularia), and suggested the two species may be congeneric. Subsequently, Gordon (1989) observed and illustrated Japanese material of *Buchneria* present in the Natural History Museum London (NHMUK), and elucidated the umbonuloid frontal shield; he concluded *Buchneria* cannot be grouped together with *Osthimosia* and other lepraliomorphs. Unfortunately, I have never had a chance to check the type material of Harmer’s *Buchneria sinuata*, which is not in NHMUK (Mary Spencer Jones, pers. comm. 11 May 2012) and both institutes Zoological Museum Amsterdam (ZMA) and Naturalis Biodiversity Center in Leiden (Elly Beglinger, pers. comm. 19 September 2012). Although I have not checked the type material, I exclude Harmer’s *Buchneria sinuata* from the description of the genus in this paper based on the significant differences with the type species of the genus; *Buchneria sinuata* is also different from other *Buchneria* species in mainly imperforate frontal shield, small colony size, and preference of unstable substrate which is unusual for *Buchneria* species. The status of *Buchneria sinuata* is still unclear and should be clarified in future work. With the removal of nominal *Buchneria sinuata* from *Buchneria*, the cormidial orifice and the rounded mandible of suboral avicularia may be considered diagnostic characters for *Buchneria*. Buchner (1924) described large frontal avicularia, but these are enlarged suboral avicularia; therefore, absence of a large frontal avicularium may also be considered diagnostic for *Buchneria*.

Gordon (1989) regarded *Buchneria* close to *Celleporaria* in Lepraliellidae, based on the similarities between the type species of both genera in the umbonuloid and imperforate frontal shield with marginal areolar pores, broad orifice, and suboral avicularium. However, the ovicell of *Buchneria* is different from that of Lepraliellidae in having broader proximal window and deeper ooecium, and is more similar to that of *Palmiskenea* Bishop & Hayward, 1989 in Bryocryptellidae with a few small foramina close to the proximal margin. *Buchneria* also resembles *Palmiskenea* in the frontal shield having only marginal areolar pores, but differs from the latter in the orifice without condyles and in polymorphic avicularia. *Buchneria* resembles *Marguetta* Jullien, 1903 in having only marginal pores, an ovicell with a few small pores, and oval suboral avicularia, but differs from the latter in lacking frontal avicularia on margin of the frontal shield. *Buchneria* also resembles *Porella* Gray, 1848 and *Porelloides* Hayward, 1979 in having only marginal pores, an ovicell without or with a few small pores, and suboral avicularia, but differs from the latter two genera in lacking lyrula and condyles. Although some species of *Porella* also lack a lyrula, and species of *Porelloides* normally lack condyles, a small tooth on the distal margin of the suboral avicularium is characteristic of *Buchneria*. Considering the similarities of ovicell and orifice morphology between *Buchneria* and the four bryocryptellid genera, *Porella*, *Palmiskenea*, *Marguetta* and *Porelloides*, I conclude *Buchneria* is much better placed in Bryocryptellidae rather than Lepraliellidae.

Harmer (1957) suggested that *Haswellia auriculata* Busk, 1884 be placed in *Buchneria*, on the basis of orifice morphology and the small lateral avicularia on the edge of the peristome. He also suggested that the species *Haswellia auriculata* and *Myriozoum marionense* Busk, 1884 as described by Calvet (in Jullien and Calvet 1903) represent a single species referable to *Buchneria*, on the basis of very few frontal pores; a large, spatulate frontal avicularium; the form of the peristome with small avicularia; and immersed ovicells (Harmer, 1957). However, *Haswellia auriculata* is currently regarded as a junior synonym of *Galeopsis pentagonus* (d’Orbigny, 1842), and the specimen described as *Myriozoum marionense* in Jullien and Calvet (1903) is also considered as a species of *Galeopsis*.

Excluding nominal *Buchneria sinuata*, *Buchneria* presently contains three species, which I redescribe here.

#### Buchneria teres

(Ortmann, 1890) comb. n.

http://species-id.net/wiki/Buchneria_teres

Figures 4 , 5 , 6

Escharoides teres Ortmann, 1890, 43, pl. 3, fig. 21; type locality, Sagami Bay.
Palmicellaria ortmanni Buchner, 1924, 210, fig. V; mentioned only in a caption to a text figure and presumably a *nomen nudum*; see Harmer (1957: 876).
Palmicellaria dofleini Buchner, 1924, 210, figs. F, V, pl. 17, figs. 9−12.
Buchneria dofleini : Harmer 1957, 876, pl. 17, figs. 9−12; Gordon 1989, 258, figs. 17−20.

##### Material examined.

*Lectotype*. Branched colony (MZS 36-2), collected by L. Döderlein, 1882, Sagami Bay. *Paralectotype*. Branched colony (MZS 36-1, 36-3; NSMT Te-738), collected by L. Döderlein, 1882, Sagami Bay. *Other material examined*. Fragment of colony ZSM 20043001, collected by F. Doflein, 17 October 1904, entrance of Tokyo Bay, 600 m depth; fragment of colony ZSM 20100261 collected by F. Doflein, 1904−1905, Sagami Bay; single small living colony on pebble and several fragments of living colonies (NSMT TeS-3, TeS-2), collected by NSMT from RV *Shinyo-maru*, 24 October 2003, Okinose, Sagami Bay (34°58.80'N, 139°31.50'E to 34°59.20'N, 139°31.20'E), 900−950 m depth, by dredge; fragments of colonies (NSMT TeS-4), collected by NSMT from research boat *Rinkai-maru*, 16 March 2001, SW of Hayama, Sagami Bay (35°11.46'N, 139°28.71'E to 35°11.64'N, 139°28.14'E), 432−580 m depth, by dredge; several living and dead colonies (NSMT TeS-5 to TeS-12) on dead *Conchocele bisecta* (Conrad, 1849) shells, collected by NSMT from RV *Tansei-maru*, 24 November 2007, ENE of Hatsushima, Sagami Bay (35°03.41'N, 139°12.55'E to 35°02.73'N, 139°13.73'E), 563−756 m depth, by beam trawl; single small dead colony on pebble (NSMT Te-876), collected by Nagai, 24 April 1997, SW of Shionomisaki, Wakayama Prefecture (33°24.91'N, 135°38.69'E to 33°24.95'N, 135°38.12'E), 500 m depth, by dredge.

##### Measurements.

ZL, 0.595−1.334 (0.978±0.160); ZW, 0.214−0.840 (0.450±0.133); n=65. OrL, 0.131−0.208 (0.171±0.021); OrW, 0.123−0.219 (0.187±0.018); n=32. AvL, 0.078−0.198 (0.110±0.017); AvW, 0.055−0.148 (0.086±0.016); n=82. OvL, 0.205−0.391 (0.311±0.049); OvW, 0.286−0.439 (0.365±0.043); n=29. Additional measurements: large suboral avicularium (LAv) length, 0.619−0.766 (0.682±0.076); LAv width, 0.398−0.453 (0.426±0.027), n=3.

##### Description.

**Figure 4.**
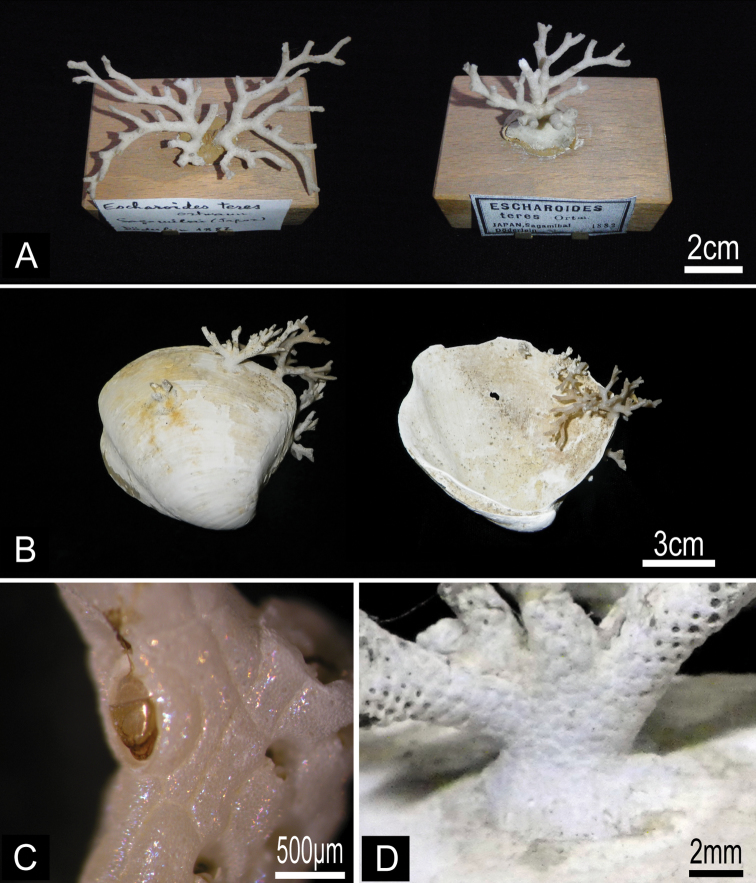
*Buchneria teres* comb. n. **A** Lectotype (MZS 36-2) and paralectotype (MZS 36-1) **B** Colonies on dead shells of *Conchocele bisecta*, NSMT TeS-8 and 10 **C** Large suboral avicularium with a semicircular mandible, NSMT TeS-2 **D** Kenozooidal base of the colony, NSMT TeS-8.

**Figure 5.**
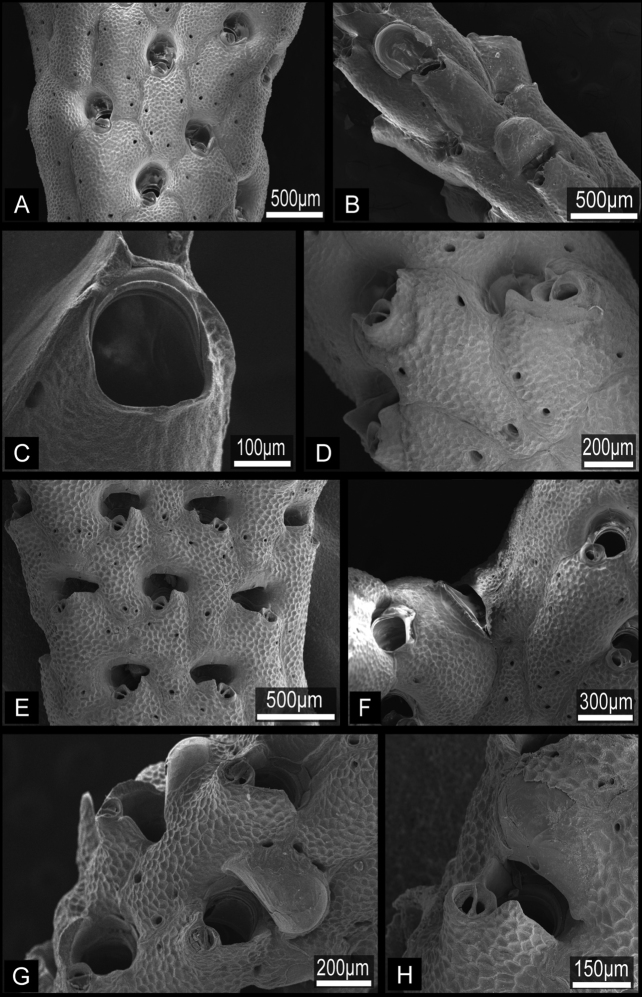
*Buchneria teres* comb. n., scanning electron micrographs. **A** Part of a branch showing autozooids with few frontal pores, NSMT TeS-2 **B** Younger part of a paralectotype branch showing rectangular zooids and semicircular ovicells, NSMT Te-738 (original MZS 36-3) **C** Orifice without lyrula or condyles, NSMT TeS-6 **D** Autozooid (right) with a developing peristomial labium of an intramural bud, NSMT TeS-3 **E** Ovicellate zooids with well-developed peristomial labia, ZSM 20100261 **F** Large avicularium at a branch bifurcation, NSMT TeS-2 **G** Zooids with young ovicells, NSMT TeS-6 **H** Ooecium partly covered by secondary calcification from surrounding zooids, ZSM 20100261.

**Figure 6.**
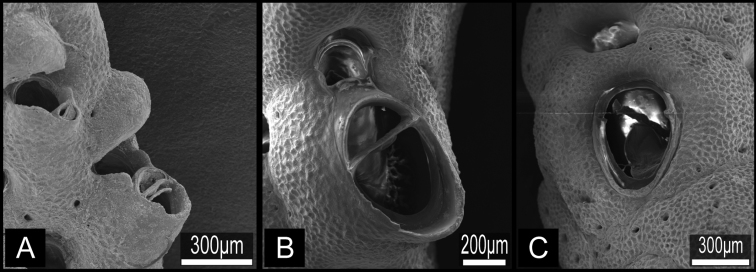
*Buchneria teres* comb. n., suboral avicularia. **A** Two different sizes of suboral avicularia in adjacent zooids, ZSM 20043001 **B** A large, projecting suboral avicularium, NSMT TeS-2 **C** A large suboral avicularium appearing offset to the center of the frontal shield, NSMT TeS-2.

Colony erect, rigid, dichotomously branching, widely spreading, antler-like, terminal branches slender. Basal part of colony composed of both autozooids and kenozooids. Branches cylindrical, 1.39–4.76 mm wide (2.77±0.85 mm; n=25), with zooids opening all around, four or five zooids across in half-view (Fig. 4A, B). Autozooids subrectangular to oval, tapering proximally, cylindrical in younger ends of branches, arranged in quincunx; zooidal borders indistinct. Frontal shield convex, entirely tessellated with minute depressions, with two to six areolar pores offset from margin (Fig. 5A, B). Orifice (Fig. 5C) deeply immersed, elongated semicircular, about as wide as long, slightly concave proximally; lyrula and condyles absent. Oral spines lacking. Secondary orifice cormidial, bounded by contributions of secondary calcification from distal and lateral zooids, with suture lines often evident between the sectors; secondary orifice roughly oval in young zooids, complex in mature zooids, with suboral avicularium offset to one side and a sharp, raised flattened peristomial flange on the other, often with a sinus between the two (Fig. 5D, E). Suboral avicularium lies on peristome periphery; small, circular, with complete pivot; semicircular mandible directed proximolaterally (Fig. 5D, H); orificial side of rostrum with a rounded-triangular tooth or flange (Fig. 5E). Zooids commonly have the small suboral avicularium replaced by a larger (Fig. 6A) or much larger, hypertrophied oval (Fig. 6B) avicularium (Fig. 6A, B), with the latter type sometimes displaced proximally toward the center of the frontal shield (Figs 4C, 6C). Another type of large avicularium occurs rarely at branch bifurcations (Fig. 5F), appearing almost as a crack in the bifurcation; twice as wide as long, 0.181 mm long by 0.373 mm wide (n=1). Interzooidal kenozooids lacking orifice are interspersed with autozooids on branches (Fig. 5A), but are often much more numerous on side of branch facing inward toward the colony axis than on outer side; kenozooids encircle the base of colony (Fig. 4D). Ovicell (Fig. 5B, G, H) globose, recumbent on distal zooid, roughly as wide as long when fully formed; ooecium smooth, proximal margin slightly curved, ectooecium is not completely covering the endooecium, leaving a large central membranous foramina and small proximal membranous window (Fig. 3A, B). Ectooecium is partially covered by tessellated secondary calcification from neighboring zooids with age (Fig. 5H). The proximal margin with the central pseudopore remains uncovered in the old parts of the colony (Fig. 3C).

##### Distribution.

Sagami Bay, Sagami Sea, Tokyo Bay, and off Kii Peninsula, at depths of 432–950 m. The collecting depth of the specimen (1921.11.7.9.) in NHMUK is 250–330 fathoms, which means 457–603 m; therefore, the depth given in Harmer (1957) is wrong and should be in feet.

##### Remarks.

Ortmann (1890) first described *Buchneria teres* (as *Escharoides teres*) based on Döderlein’s specimens from eastern Sagami Bay; these specimens had not been reexamined until this study. Buchner (1924) subsequently described *Buchneria dofleini* (as *Palmicellaria dofleini*) from Doflein’s Sagami Bay specimens. Although Buchner’s type specimen of *Buchneria dofleini* was lost during WWII, I found other specimens he identified as this species in the Doflein collection at ZSM. In comparing these specimens with Ortmann’s syntypes of *Escharoides teres*, I found no diagnostic differences between the two; Buchner’s specimens are simply the distal younger part of branches of *Escharoides teres*. I thus consider *Buchneria dofleini* as a junior synonym of *Escharoides teres*, which accordingly becomes the type species of *Buchneria*. Buchner (1924) reported large frontal avicularia in his species, but these are enlarged suboral avicularia. Although syntypes of *Buchneria teres* in the Döderlein collection lack the colony base, I found an entirely kenozooidal colony base in complete colonies recently collected from Sagami Bay (Fig. 4D).

#### Buchneria rhomboidalis

(Ortmann, 1890) comb. n.

http://species-id.net/wiki/Buchneria_rhomboidalis

Figures 7 , 8

Escharoides rhomboidalis Ortmann, 1890, 44, pl. 3, fig. 22; type locality, eastern Sagami Bay.

##### Material examined.

*Lectotype*. MZS 37-2 (NSMT Te-737), branched colony, collected by L. Döderlein, 1882, Sagami Bay. *Paralectotype*. MZS 37-1, branched colony, collected by L. Döderlein, 1882, Sagami Bay, 370 m depth. *Other material examined*. NSMT-Bry R256, Emperor Showa Collection, collected 8 February 1967, 5 km SW of Jogashima, Sagami Bay, 250–400 m depth; NSMT-Bry R267, Emperor Showa Collection, collected 18 March 1968, 4 km WSW of Jogashima, Sagami Bay, 200−220 m depth; NSMT TeS-1, coll. 14 May 2004, west of Ōshima, Sagami Sea (34°40.95'N, 139°17.92'E to 34°40.68'N, 139°18.22'E), 220−277 m depth, beam trawl, RV *Tansei-maru*; colony (NSMT Te-799), collected by H. Kohtsuka from research boat *Rinkai-maru*, 10 January 2012, SW of Jogashima, Sagami Bay (35°06.101'N, 139°34.284'E to 35°05.684'N, 139°34.061'E), 218–318 m depth; fragments of colonies (NSMT Te-796, Te-797), collected by M. Hirose from research boat *Rinkai-maru*, 24 February 2012, WSW of Jogashima, Sagami Bay (35°07.301'N, 139°33.365'E to 35°07.327'N, 139°32.978'E), 300–493 m depth.

##### Measurements.

ZL, 0.767−1.150 (0.948±0.100); ZW, 0.468−1.050 (0.735±0.116); n=32. OrL, 0.152−0.211 (0.177±0.019); OrW, 0.189−0.245 (0.222±0.023); n=14. AvL, 0.106−0.271 (0.171±0.034); AvW, 0.096−0.193 (0.144±0.024); n=28.

##### Description.

**Figure 7.**
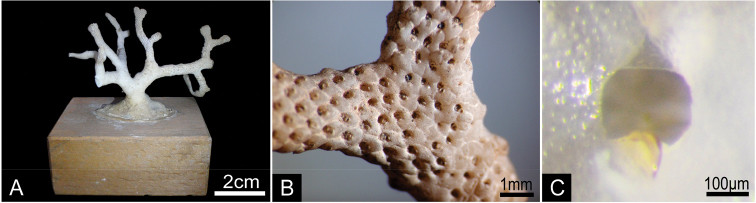
*Buchneria rhomboidalis* comb. n. **A** Lectotype in the Döderlein collection, MZS 37-2 **B** Enlargement of a branch of the lectotype **C** Enlargement of an orifice showing the proximal margin of the ooecium, NSMT Te-796.

**Figure 8.**
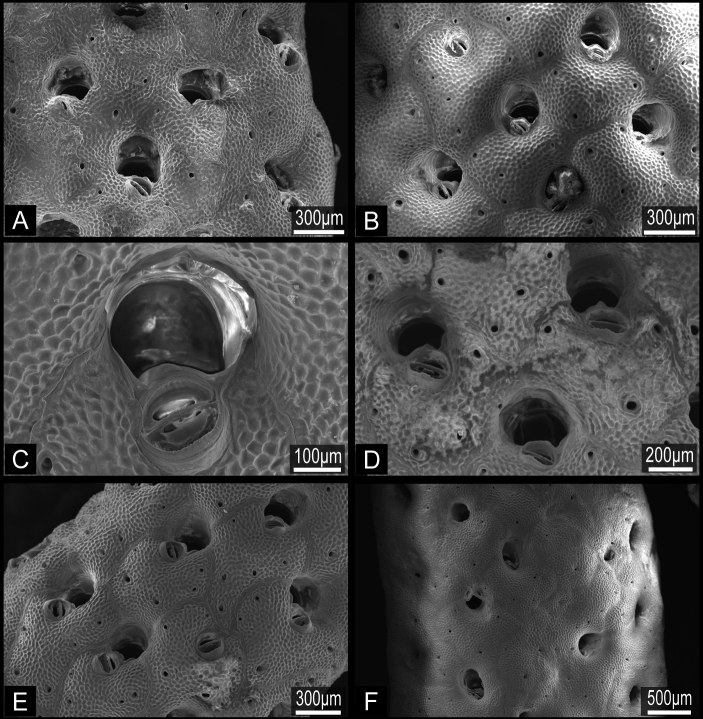
*Buchneria rhomboidalis* comb. n., scanning electron micrographs. **A** Part of the lectotype, NSMT Te-737 (original MZS 37-2) **B** Specimen NSMT-Bry R256, showing rhomboidal autozooids with few frontal pores **C** Orifice with a round suboral avicularium, NSMT-Bry R267 **D** Suboral avicularia, each with a small, conical distal tooth, NSMT-Bry R267 **E** Autozooids, showing the slightly projecting rostrum of the suboral avicularia, NSMT-Bry R267 **F** Kenozooids interspersed with autozooids at the base of the colony, NSMT TeS-1.

Colony erect, rigid, dichotomously branching, widely spreading (Fig. 7A). Branches flattened, multiserial, with zooids opening all around; 2.33−6.34 mm wide (3.34±0.88 mm, n=25), five to nine zooids across (Fig. 7B). Autozooids rhomboidal, arranged in quincunx (Fig. 7B), zooidal borders indistinct. Frontal shield convex, entirely tessellated with minute depressions, with two to four small areolar pores (Fig. 8A, B) offset from margin. Orifice subcircular, about as wide as long, smooth distally, proximal margin without sinus; lyrula and condyles absent (Fig. 8C). No oral spines. Orifice deeply immersed; aperture at colony surface roughly semicircular in outline, without sinus proximally; cormidial, bounded by contributions of frontal calcification from distal and one or two lateral zooids, with suture lines sometimes evident between the sectors (Fig. 8C, D). Suboral avicularium small, proximal to orifice on the peristome periphery; circular, with complete pivot, rostrum slightly elevated, slightly denticulate, semicircular mandible directed proximolaterally (Fig. 8C, D, E); distal tooth of suboral avicularium small, rounded-conical (Fig. 8D). No other avicularia were observed. On both the edges of branches and in older part of colony, interzooidal kenozooids lacking orifice (Fig. 8F) are interspersed with autozooids; kenozooids especially numerous in the basal part of colony. Ooecium imperforate, smooth, completely immersed, not evident from colony surface; proximal margin almost straight or slightly curved, rarely obscuring the distal edge of primary orifice in oviccelate zooids (Fig. 7C).

##### Distribution.

Eastern part of Sagami Bay, and the Sagami Sea southwest of Jogashima and west of Ōshima, at depths of 200–493 m.

##### Remarks.

Examination of Ortmann’s (1890) type specimens revealed this species belongs not in *Escharoides* but in *Buchneria*, on the basis of the frontal shield with few pores, absence of oral spines, orifice without lyrula, immersed imperforate ooecium, and the suboral avicularia. *Buchneria rhomboidalis* is characterized by having rhomboidal zooids and flat branches. This species resembles *Buchneria teres*, but differs in having flat rather than cylindrical branches, in lacking a peristomial labium and sinus, and in lacking a large avicularium at branch bifurcations. The depth distribution of *Buchneria rhomboidalis* (200–493 m) is shallower than that of *Buchneria teres* (432–3660 m).

#### Buchneria variabilis

(Androsova, 1958) comb. n.

http://species-id.net/wiki/Buchneria_variabilis

Figures 9 , 10

Porella variabilis Androsova, 1958, 165, fig. 96; type locality, Moneron Island, northern Sea of Japan.

##### Material examined. 

Androsova’s type specimen (ZIN-1/3670) in Zoological Institute of the Russian Academy of Sciences (ZIN RAS), colony collected southwestern region of Sakhalin, Moneron Island (Kaibato), Sea of Japan, 36 m depth, (examined by micrographs); large erect colonies and fragments (NSMT Te-724 to Te-734; ZIHU 4130 and 4131), collected SE of Akkeshi Bay (42°48.37'N, 144°56.22'E) by M. Hirose from research boat *Misago-maru*, 6 July 2010, 116 m depth, by dredge; large erect colony and fragments (NSMT Te-790 to Te-794) collected SE of Akkeshi Bay (42°48.20'N, 144°55.43'E to 42°48.26'N, 144°54.91'E) by M. Hirose from research boat *Misago-maru*, 8 July 2011, 114−116 m depth, by dredge.

##### Measurements.

ZL, 0.558−0.921 (0.751±0.101); ZW, 0.408−0.882 (0.611±0.088); n=25. OrL, 0.135−0.223 (0.189±0.019); OrW, 0.130−0.226 (0.192±0.023); n=27. OvL, 0.124−0.444 (0.247±0.072); OvW, 0.104−0.395 (0.214±0.056); n=44.

##### Description.

**Figure 9.**
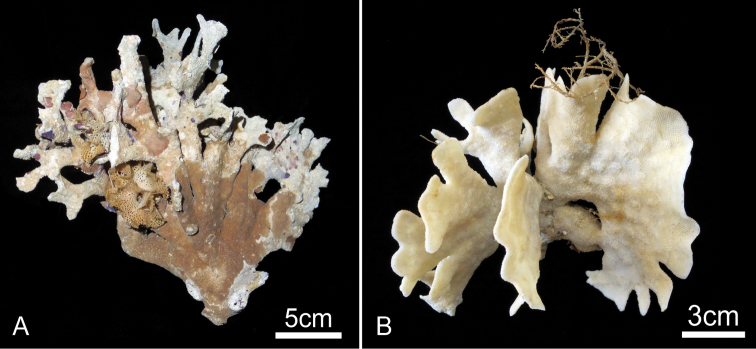
*Buchneria variabilis* comb. n. **A** Large, bushy dead colony with various encrusting epibionts, NSMT Te-726 **B** Large, fan-shaped living colony, NSMT Te-724; note the broad circular monticules at centre right.

**Figure 10.**
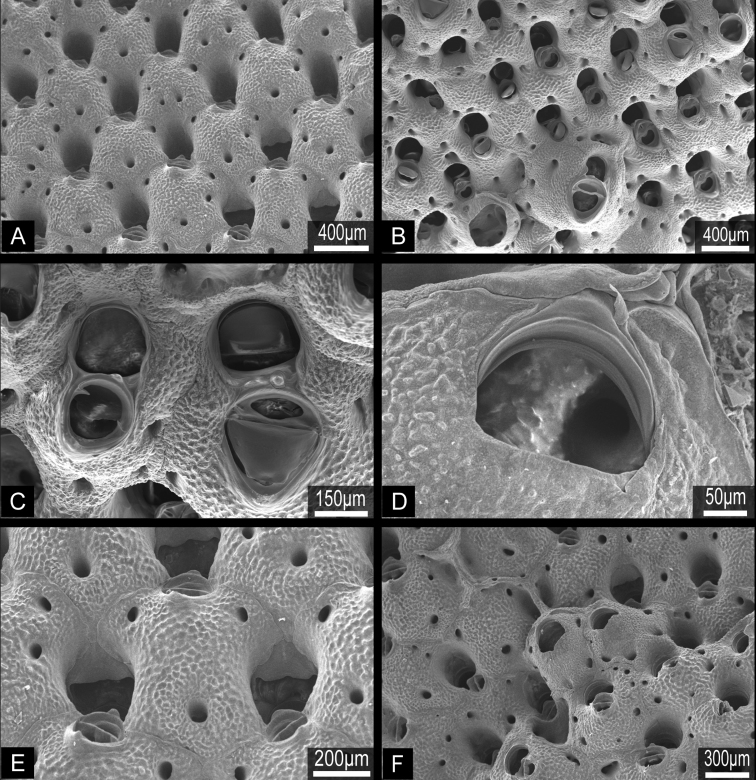
*Buchneria variabilis* comb. n., NSMT Te-729, scanning electron micrographs. **A** Autozooids showing the strongly convex frontal wall with few pores **B** Autozooids with various sizes of suboral avicularia **C** Adjacent autozooids showing different sizes of suboral avicularia **D** Orifice without lyrula or condyles **E** Enlargement showing immersed orifices and the narrow, conical tooth distal to suboral avicularia **F** Overgrowth by frontal budding in the central part of the colony.

Colony erect, rigid, robust, with thick, broad, strap-like branches at least 10 zooid widths across, or foliaceous, fan-shaped lobes; lobes or branches 0.86 to 8.04 cm wide (2.15±1.38 cm, n=25), multifurcate or irregularly lobed on distal margin; zooids open on both sides (Fig. 9). Broad lobes of some colonies are covered with conspicuous, closely spaced circular monticules (Fig. 9B). Autozooids oval, rounded hexagonal, or subrectangular in outline; strongly convex frontally, arranged in quincunx, zooecial borders indistinct; frontal shield tessellated, with four to eight areolar pores of irregular size along margin or offset more centrally (Fig. 10A, B). Orifice (Fig. 10C, D) semicircular, broader than long, slightly concave proximally, lyrula and condyles absent (Fig. 10D); deeply immersed with age. Oral spines lacking. Peristome deep, cormidial, formed by contributions of secondary calcification from distal and lateral zooids, with suture lines often evident between the sectors (Fig. 10E). Suboral avicularia approximately same size as orifice, located at margin of peristome; oval, with complete or incomplete pivot, rostrum slightly elevated distally, with a median tooth; mandible semicircular, directed proximally or proximolaterally (Fig. 10B, C). Rounded conical tooth on oral edge of avicularian rostrum conspicuous, projecting into secondary orifice (Fig. 10E). Hypertrophied suboral avicularia are frequent; often larger in area than orifice; distal end of rostrum elevated, pointed; rounded-triangular mandible directed proximally (Fig. 10B, C). I observed no other types of avicularia. Basal part of colony robust, composed of both interzooidal kenozooids and autozooids, borders indistinct. Ooecium imperforate, smooth, completely immersed by secondary calcification from the neighboring zooids, the proximal margin of the ooecium distinctly indented laterally and centrally, obscuring the distal edge of primary orifice in ovicellate zooids (Fig. 10E). Frontal budding frequent (Fig. 10F).

##### Distribution.

Moneron Island, SW Sakhalin, 36 m depth (Androsova 1958); off Akkeshi Bay, Hokkaido, 114–116 m depth (this study).

##### Remarks.

My material matches Androsova’s (1958) description of *Porella variabilis*. She mentioned that the tooth on the proximal margin of the peristome is associated with the suboral avicularium, and that the tooth is present when the avicularium abuts the proximal margin of the peristome, but absent when the avicularium is offset proximally from the peristome. This is the case in all known species of *Buchneria*. Androsova (1958) reported *Buchneria variabilis* from 36 m depth, shallower than my specimen from 114–116 m near Akkeshi. This apparent difference in the bathymetric distribution between Sakhalin and Akkeshi may be related to water temperature, as Sakhalin is more northern and colder than Akkeshi.

*Buchneria variabilis* differs from *Buchneria teres* and *Buchneria rhomboidalis* in colony form and in having larger suboral avicularia.

## Discussion

**Figure 11.**
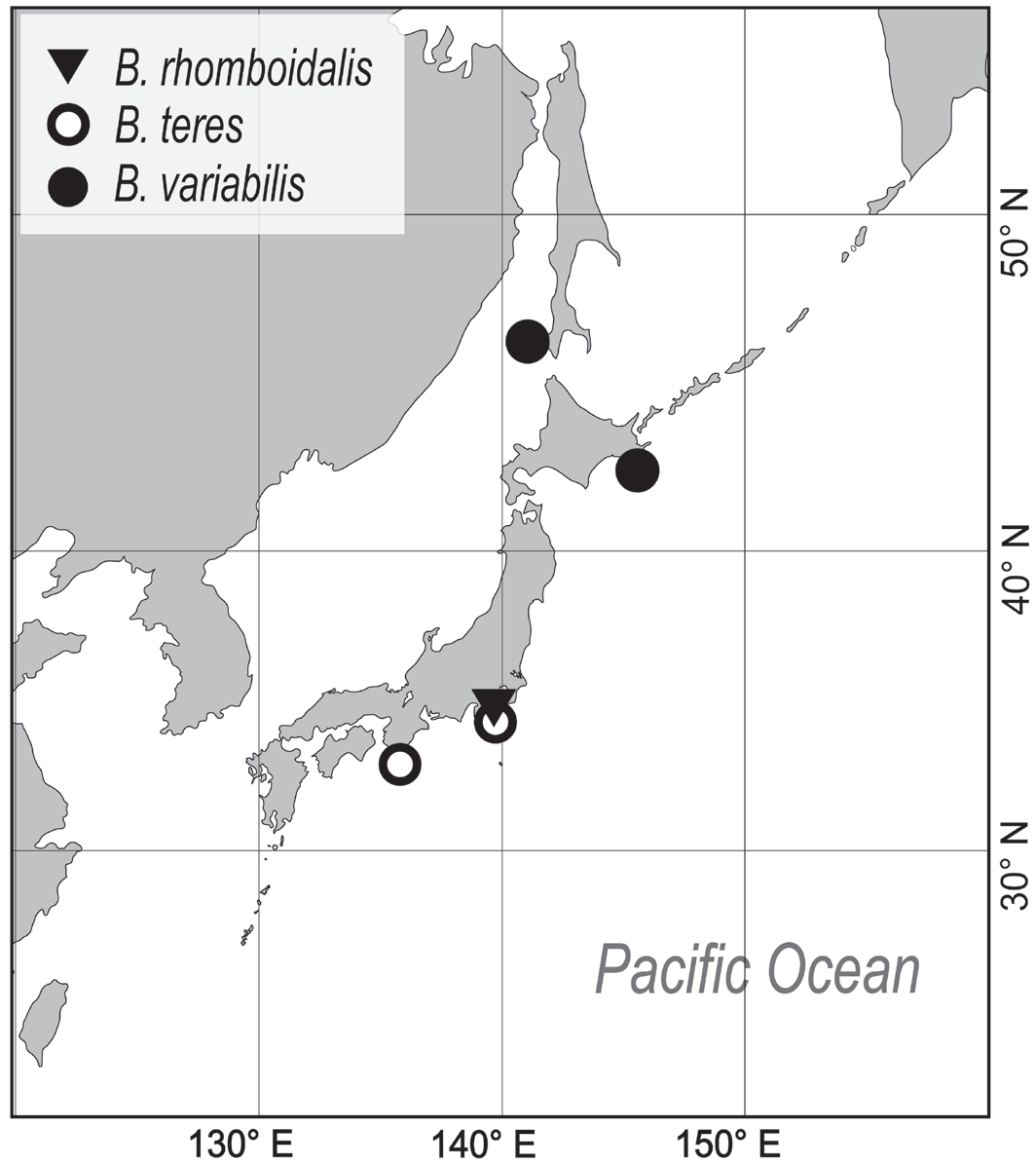
Map showing the known distribution of the three *Buchneria* species in the western Pacific.

To date, species of *Buchneria* have been only reported from the northwestern Pacific, where they appear to have a cold-temperate distribution (Fig. 11). Most records are from northern Japanese waters; I have not detected *Buchneria* species in field surveys in southern Japan (e.g., near Okinawa). The southernmost record of living *Buchneria* in Japan is *Buchneria rhomboidalis* and *Buchneria teres* from Sagami Bay, of which *Buchneria teres* has been considered as abyssal species (Buchner, 1924; Harmer, 1957) and was collected at depths of more than 400 m in Sagami Bay. Another, more northern *Buchneria* species in Japan showed shallower distribution; *Buchneria variabilis* occurred at 114–116 m depth near Akkeshi and at 36 m depth near Sakhalin (Androsova, 1958).

While *Buchneria* might be endemic to this region, further sampling around the North Pacific rim, including deep-water sites, may expand the distributions of known species or detect additional species. Furthermore, as *Buchneria* resembles some other North Atlantic genera such as *Porella*, *Palmiskenea*, *Marguetta* and *Porelloides*, taxonomic studies of these other genera may also detect additional *Buchneria* species, and the close relationship may indicate a common history at times when there was a connection between the Pacific and Atlantic.

### Taxonomic key to Japanese *Buchneria* species

**Table:** 

1a	Colony robust; branches thick and broad, fan-shaped distally; hypertrophied suboral avicularia often occur on the frontal shield	*Buchneria variabilis* comb. n.
1b	Colony more delicate, branches cylindrical or flattened, slender distally; hypertrophied suboral avicularia rare or absent	2
2a	Branches flattened; zooids rhomboidal; hypertrophied suboral avicularia absent	*Buchneria rhomboidalis* comb. n.
2b	Branches cylindrical; zooids rectangular, hypertrophied suboral avicularia present	*Buchneria teres* comb. n.

## Supplementary Material

XML Treatment for Buchneria

XML Treatment for Buchneria teres

XML Treatment for Buchneria rhomboidalis

XML Treatment for Buchneria variabilis

## Appendix

Specimens examined in this study. (doi: 10.3897/zookeys.241.3175.app File format: Microsoft Office Excel file (xls) **Explanation note:** The deposit column indicates the museum where specimens reside: MZS, Musée Zoologique Strasbourg, ZSM, Zoologische Staatssammlung München, NSMT, National Museum of Nature and Science Tokyo (now located in Tsukuba).
